# La mastite granulomateuse idiopathique: à propos de 4 cas et revue de littérature

**DOI:** 10.11604/pamj.2020.37.128.25301

**Published:** 2020-10-05

**Authors:** Hind Ennasser, Jamal Eddine Raoudi, Hafsa Taheri, Hanane Saadi, Ahmed Mimouni

**Affiliations:** 1 Service de Gynécologie-Obstétrique, Centre Hospitalier Universitaire Mohammed VI Oujda, Oujda, Maroc

**Keywords:** Mastite granulomateuse, nodules giganto-épithélio-cellulaires, nécrose caséeuse, granulome tuberculoïde, Granulomatous mastitis, giant-epithelial-cell nodules, caseous necrosis, tuberculoid granuloma

## Abstract

La mastite granulomateuse idiopathique est une mastopathie bénigne inflammatoire rare. L´examen histologique d´une biopsie chirurgicale permet de confirmer le diagnostic et écarter ainsi une mastite carcinomateuse. Bien que d’étiologies indéterminées et survenue surtout chez la femme jeune, son caractère idiopathique est retenu après élimination des autres causes de la maladie granulomateuse. Le traitement médical repose sur l´antibiothérapie et les anti-inflammatoires. Il est chirurgical dans les formes compliquées. Nous rapportons quatre cas de mastite granulomateuse idiopathique colligés au sein du Service de Gynécologie et Obstétrique de l´Hôpital Universitaire MED VI d´Oujda, Maroc.

## Abstract (en)

Idiopathic granulomatous mastitis is a rare benign inflammatory mastopathy. Histological examination of the surgical biopsy can confirm the diagnosis and thus exclude carcinomatous mastitis. Although its etiologies are undeterminated and it occurs mainly in young women, its idiopathic nature is confirmed after exclusion of the other causes of granulomatous disease. Medical treatment is based on antibiotic therapy and anti-inflammatory drugs. Patients with complicated forms are treated with surgery. We here report four cases of idiopathic granulomatous mastitis whose data were collected in the Department of Gynecology and Obstetrics at the University Hospital MED VI of Oujda (Morocco).

## Introduction

La mastite granulomateuse est une pathologie rare, décrite pour la première fois en 1972 par Kessler and Wolloch [1]. L´aspect macroscopique et radiologique des lésions mammaires orienterait vers une origine carcinomateuse. Le diagnostic de certitude est histologique et son évolution reste capricieuse avec risque de récidives. Notre analyse porte sur quatre cas, tout en élucidant la donnée clinique, paraclinique et anatomopathologique.

## Patient et observation

**Figure 1:**
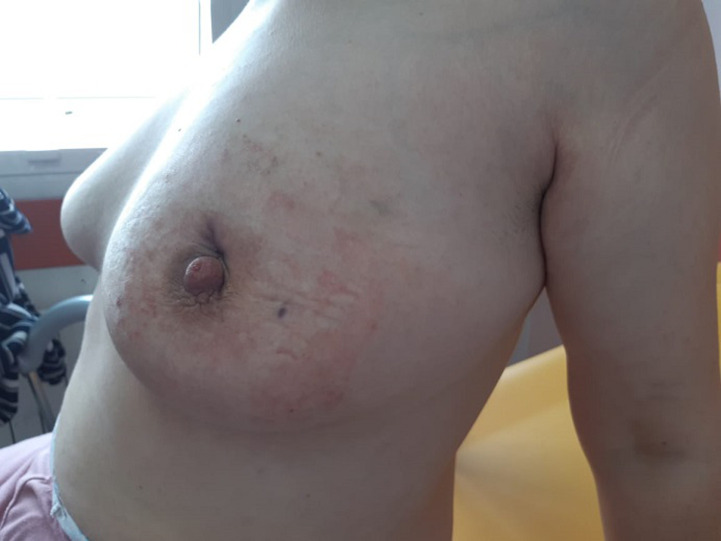
aspect macroscopique du sein gauche montrant l´évolution favorable après drainage chirurgical et traitement médical associant antibiotiques et anti-inflammatoires

**Observation 1:** Mme R.B âgée de 32 ans, sans antécédents pathologiques notables, mère d´un enfant vivant et qui présente au niveau du sein gauche une tuméfaction retromamelonnaire mesurant 5 cm, dure indolore et adhérente au plan superficiel, avec une rétraction mamelonnaire en regard. La lésion est classée T4b N0 Mx. Le sein controlatéral est sans particularité avec des aires ganglionnaires libres. L´écho-mammographie montre une masse retromamelonnaire à contenu liquidien impure et impropre de 30 mm. Elle est associée à des micro-calcifications, à un épaississement diffus du tissu cutané et sous cutané, et à une rétraction mamelonnaire. On note également la présence de multiples adénopathies axillaires dont la plus grande mesure 16 mm. La lésion est classée ACR4. L´examen histologique d´une microbiopsie au Tru-cut confirme la mastite granulomateuse. L´évolution est marquée par une abcédation en voie de fistulisation. La patiente a bénéficié d´un drainage d´abcès avec une biopsie chirurgicale dont l´étude anatomopathologique a révélé la présence de cellules géantes et épithéloïdes groupées en formations nodulaires au centre nécrotique, réalisant un aspect de mastite granulomateuse. Les suites postopératoires, après 4 jours d´hospitalisation, sont favorables sous AINS (Figure 1). Le bilan étiologique est revenu normal (sérologies virales HVC HVB VIH, sérologie syphilitique, TDM TAP, bilan phosphocalcique, dosage de l´enzyme de conversion de l´angiotensine, auto-anticorps, dosage du complément sérique, quantiferon) confirmant la mastite granulomateuse idiopathique.

**Figure 2:**
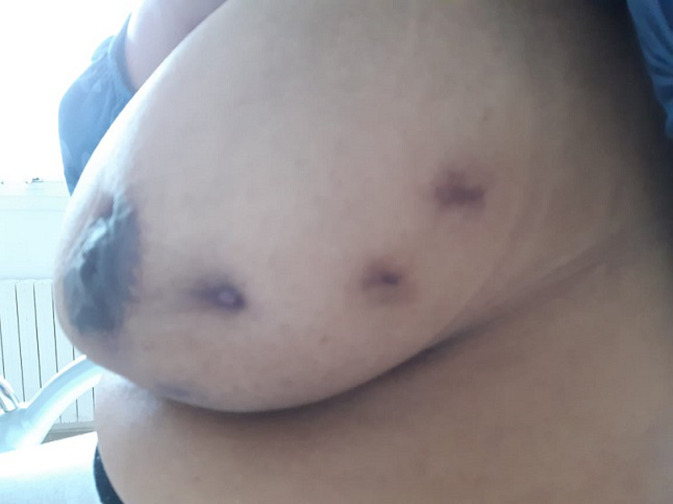
aspect macroscopique du sein gauche montrant l´évolution favorable après drainage chirurgical et traitement anti inflammatoire, avec persistante des cicatrices d´orifices de fistulisation

**Observation 2:** Mme B.F, âgée de 44 ans, mère d´un enfant vivant, ayant comme antécédent familial une néoplasie mammaire du 2^e^ degré et qui consulte pour la découverte fortuite depuis 2 mois à l´autopalpation d´un nodule du sein gauche de 4cm indolore, mobile par rapport aux deux plans, à la jonction des quadrants externes, classé T2N0Mx. Les aires ganglionnaires sont libres. L´écho-mammographie montre un infiltrat hyperéchogène de contours mal limités, associé à des adénopathies axillaires pathologiques dont la plus grande mesure 17 mm. La lésion est classé ACR 4. La micro biopsie au Tru-cut est en faveur d'une mastite subaiguë et granulomateuse. L´évolution est marquée par une augmentation du volume mammaire avec installation d´une mastodynie. L´examen clinique trouve une masse mammaire, occupant tout le sein gauche, mesurant 9 cm de grand axe, dure, mobile par rapport aux deux plans, associée à des signes inflammatoires et une rétraction mamelonnaire. On note également une fistule cutanée avec issue du pus et une adénopathie axillaire gauche de 1 cm mobile et douloureuse. La lésion est classée T4bN0Mx. L´échographie mammaire montre deux collections hypoéchogènes hétérogènes du tissu glandulo-graisseux, mesurant 46/11 mm et 23/12 mm. L´une est fistuliseé siégeant au niveau de la jonction des quadrants externes, et l´autre en retromamelonnaire. On note également des adénopathies axillaires pathologiques. La patiente a bénéficié d´un drainage chirurgical de l´abcès avec réalisation de multiples biopsies confirmant le diagnostic de mastite granulomateuse. L´examen cytobactériologique du pus est revenu stérile. Les suites postopératoires sont favorables sous antibiotiques, anti-inflammatoires et soins locaux (Figure 2). Le bilan étiologique est revenu normal.

**Figure 3:**
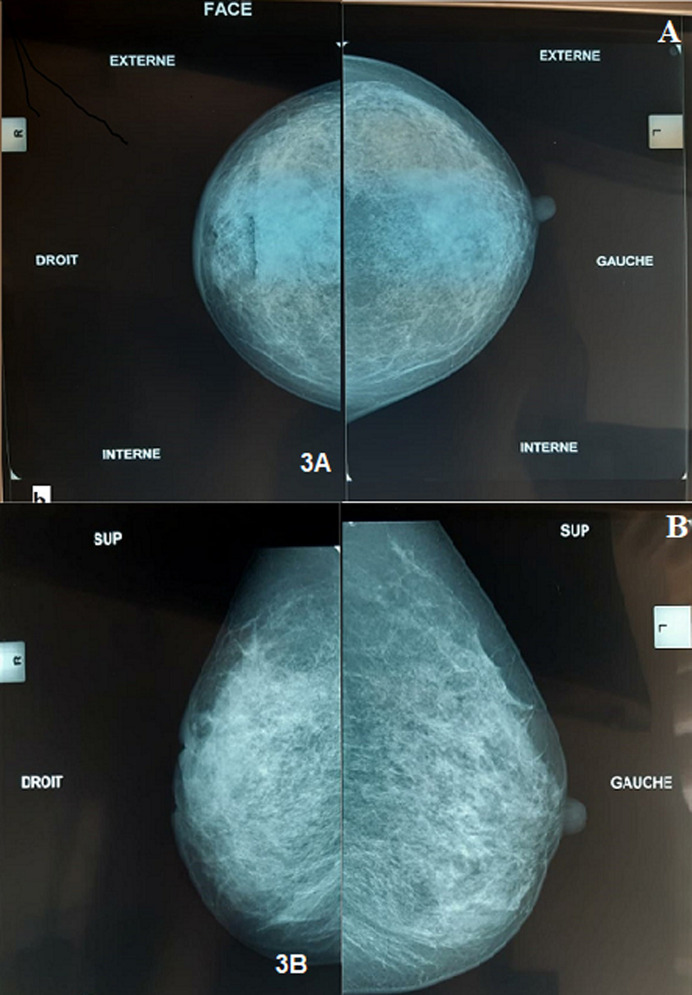
A) aspect mammographique en incidence oblique externe montrant une inégalité de taille des deux seins avec densification du sein droit et épaississement cutané; B) aspect mammographique en incidence cranio-caudale montrant une inégalité de taille des deux seins avec densification du sein droit et épaississement cutané

**Observation 3:** Mme G.O, âgée de 31 ans, G2P2, ayant comme antécédent un drainage, à deux reprises; d’un abcès mammaire droit avec de multiples biopsies, dont le résultat anatomopathologique objective un abondant matériel nécrotico-leucocytaire bordé par un tissu de granulation dense et polymorphe. L´examen cytobactériologique du pus est stérile. L´examen clinique montre une masse mammaire de 4 cm siégeant au niveau du QSE du sein droit, dure, mobile par rapport aux deux plans, fistulisée avec issue du pus. Les aires ganglionnaires sont libres. L´échographie mammaire (Figure 3) montre la présence d´une hypertrophie du parenchyme mammaire droit en rapport avec une mastite, avec une collection liquidienne ovalaire de 26*12 au niveau de la jonction des quadrants externes faisant évoquer un abcès. Un épaississement cutané a été noté. La lésion est classée ACR3. La patiente a bénéficié d´un drainage chirurgical de l´abcès avec réalisation de multiples biopsies. L´examen histologique est compatible avec un abcès mammaire comportant des follicules épithélioides microscopiques associés à des cellules géantes de type Langhans et sans nécrose caséeuse. Les suites postopératoires sont favorables sous traitement. Le bilan étiologique est revenu normal.

**Figure 4:**
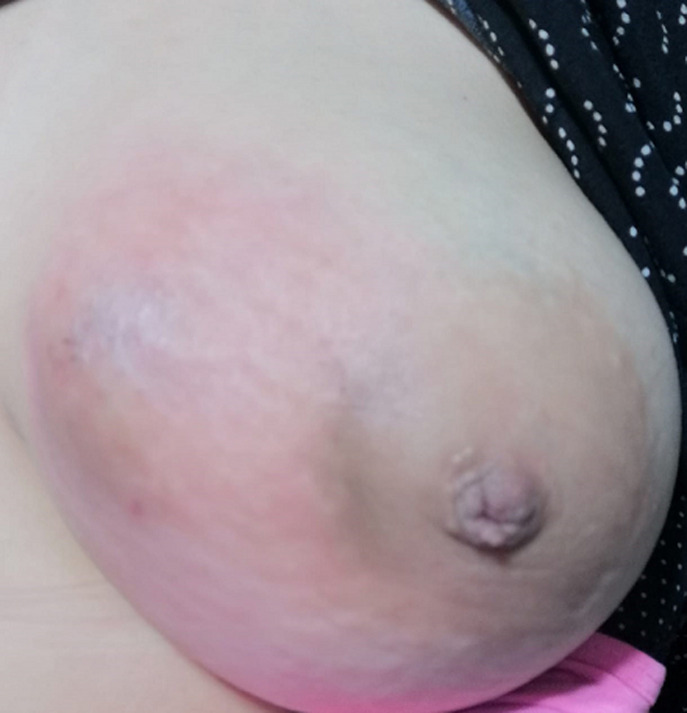
aspect macroscopique du sein gauche montrant une tuméfaction du sein gauche révélant une mastite granulomateuse

**Observation 4:** Mme A.B, âgée de 34 ans, sans antécédents pathologiques notables. Ayant présenté il y a 3 ans une tuméfaction du sein gauche d´allure inflammatoire évoluant vers l´abcédation qui a été mise sous traitement antibiotique et anti-inflammatoire avec une évolution favorable. 3 ans plus tard, la patiente s´est présentée en consultation pour tuméfaction au niveau du même sein. L´examen clinique montre une patiente en bon état général, avec une tuméfaction du sein gauche, faisant 3 cm de diamètre, siégeant à cheval entre les 2 quadrants internes, mobile, ferme avec une rougeur en regard sans écoulement mammaire associé (Figure 4). Le sein controlatéral est normal. Par ailleurs, les aires ganglionnaires sont libres, le reste de l´examen somatique est sans particularités. La mammographie a objectivé une opacité ovalaire de 3 cm siégeant à cheval des quadrants internes classé ACR3 avec absence d´anomalies au niveau du reste du sein et le sein controlatéral. Une microbiopsie au Tru-cut retrouve des cellules géantes et épithéloïdes groupées en follicules formant des granulomes tuberculoïdes sans nécrose caséeuse centrale en faveur d´une mastite granulomateuse. La patiente était mise sous traitement antibiotique et anti-inflammatoire avec une évolution favorable.

## Discussion

La mastite granulomateuse est une mastopathie bénigne inflammatoire chronique et rare. Elle représente 0,5% des tumeurs du sein affectant les canaux galactophoriques proximaux sous aréolaires, et intéressant la femme en période d´activité génitale [1]. Elle a été décrite et nommée Mastitis obliterans, en premier temps par INGIER en 1909 [2]. En 1951, Haagensen [3] l´avait nommé maladie granulomateuse perigalactophorique. Deux théories sont incriminées pour expliquer le mécanisme étiopathogénique [4]: selon Haagensen la première cause est la dilatation galactophorique avec une stase des produits de sécrétion engendrant une rupture mécanique ou chimique de l´épithélium canalaire, et déclenchant ainsi une réaction inflammatoire; selon BOSNER c´est l´inflammation péri-canalaire qui va entrainer l´altération galactophorique suite aux lésions conjonctivales; d´autres théories ont été évoquées notamment une origine infectieuse ou une réaction à un traumatisme. Cependant, la lésion commune reste une agression de l´épithélium canalaire à l´origine d´une extravasation des secrétions glandulaires dans le tissu conjonctif du lobule responsable des lésions inflammatoires granulomateuses. Cette pathologie pourrait simuler macroscopiquement et radiologiquement une lésion néoplasique. Le diagnostic de certitude est histologique montrant une mastite granulomateuse sans nécrose caséeuse.

La présentation clinique la plus fréquente est la forme nodulaire mal limitée dont les dimensions varient entre 0.5 à 10 cm, et qui peut secondairement s´abcéder. La peau sus-jacente peut être le siège de signes inflammatoires voire fistule pour les formes abcédées. La présence des adénopathies axillaires est décrite. Parfois les mastites granulomateuses sont révélées par des abcès récidivants [5-7]. Sur le plan radiologique, la mammographie peut montrer [1, 2]: une opacité diffuse avec épaississement du revêtement cutané voire opacité nodulaire au stade inflammatoire; des opacités rubanées et linéaires en rapport avec une dilatation des canaux gactophoriques à un stade avancé. Les calcifications bénignes sont souvent notées; en échographie, Boo-Kyung Han [8] avait décrit la présence des lésions tubulaires hypoéchogènes de façon fréquente.

La biopsie reste l´examen de référence montrant des granulomes épithélioïdes sans nécrose caséeuse, associés à un infiltrat inflammatoire polymorphe, constitué de plasmocytes, de lymphocytes et de polynucléaires neutrophiles [9]. Le diagnostic différentiel se fait essentiellement avec le cancer du sein dans sa forme inflammatoire et les mastites infectieuses ou non (cytostéatonecrose, sarcoïdose, maladie de Wagner) [10]. La mastite granulomateuse ne peut être étiquetée idiopathique qu´après avoir éliminé toutes causes de mastite granulomateuse et aussi afin de ne pas négliger une chance de guérison rapide par un traitement adapté d´autant plus que c´est une pathologie chronique et trainante. Aucun consensus n´a été établit pour le traitement des mastites granulomateuses. Au stade de mastite inflammatoire, le traitement repose sur les antibiotiques et les anti-inflammatoires, cependant au stade d´abcès collecté le drainage reste le traitement de choix tout en continuant le traitement médical [2].

## Conclusion

La mastite granulomateuse est une entité peu connue, souvent confondue avec la mastite carcinomateuse. L´examen anatomopathologique des lésions confirme le diagnostic. Le traitement est médical associant antibiotiques et anti-inflammatoires. Il est chirurgical dans les formes abcédées.
